# Echocardiography-guided assessment of mouse cardiac transplant rejection improves model reproducibility

**DOI:** 10.3389/frtra.2026.1840060

**Published:** 2026-06-17

**Authors:** Angela Clemens, Sara Alibrandi, Paolo Molinari, Johan Noble, Barbara Franchin, Miguel Fribourg, Paolo Cravedi, Nicholas Chun

**Affiliations:** 1 Translational Transplant Research Center and Barbara T. Murphy Division of Nephrology, Icahn School of Medicine at Mount Sinai, New York, NY, United States; 2 Unit of Nephrology, Dialysis and Kidney Transplantation, Fondazione IRCCS Ca' Grande Ospedale Maggiore Policlinico di Milano, Milan, Italy; 3 Nephrology, Hemodialysis, Apheresis and Kidney Transplantation Department, University Hospital Grenoble, Grenoble, France; 4 Kidney and Pancreas Transplantation Unit, Department of Surgery, Oncology and Gastroenterology DISCOG, University Hospital of Padova, Padova, Italy

**Keywords:** alloimmunity, echocardiagraphy, heterotopic heart transplantation, reproducibility, T cell reactivity

## Abstract

**Background:**

The mouse heterotopic heart transplant model is a widely used system for dissecting mechanisms of graft rejection and identifying new therapeutic targets, owing to its relative technical simplicity and the availability of well-defined inbred strains. One limitation of the model is that monitoring of the non-life sustaining implanted graft by manual palpation does not provide information regarding the magnitude of the anti-donor immune response and is operator-dependent. Building on previous studies suggesting that trans-abdominal ultrasonography can detect graft rejection more precisely than manual palpation, we tested if changes in motion mode (M-mode) ultrasound also correlated with the magnitude of the anti-donor T cell response.

**Methods:**

We performed sets of MHC-mismatched heterotopic heart transplants in mice and quantified the accuracy of palpation vs. ultrasonography in determining graft rejection. In an additional set of mice, we performed mixed lymphocyte reactions to quantify anti-donor T cell immunity at a fixed time point (usual experimental design) or at time points determined by changes in allograft M-mode analysis.

**Conclusions:**

The data demonstrate that ultrasonography is superior to manual palpation for determining graft rejection and newly shows that changes in M-mode associate with the magnitude of anti-donor T cell immunity. These findings are important to increase reproducibility and translational impact of studies using this model.

## 1 Introduction

Heterotopic mouse heart transplant is the most widely adopted pre-clinical model used to study vascularized solid organ immunobiology. The model has several benefits, including relative technical ease and the generalizability of mechanistic findings to other solid organ transplants (1). However, like all experimental systems, it has inherent limitations (1, 2).

The heterotopic donor heart is typically transplanted in the abdomen (though it can also be transplanted in the groin or the neck) with the blood flow “reversed,” transiting from the recipient's aorta through the donor inferior vena cava before returning to the recipient's venous system via the donor pulmonary artery. The transplanted heart graft is not life sustaining and the chambers do not pump against physiologic pressures, but the heart is perfused through the coronary arteries and the heartbeat can be detected by manual palpation (3).

Challenges arise because the strength of the palpated heartbeat does not correlate with histologic rejection grade (4) or the magnitude of the alloimmune response (5). Accurate detection of cessation is also nuanced and difficult to standardize. These limitations lead to inconsistent rejection kinetics and necessitate that immune profiling or pharmacologic interventions be performed at pre-specified post-transplant time points rather than at the time of functional deterioration. This approach relies on the assumption that rejection occurs uniformly across all animals in a cohort, which is often not true (6).

Echocardiography has emerged as a valuable tool for assessing cardiac function in mouse models of heart disease. M-mode and Doppler echocardiography can detect left ventricular (LV) chamber dilation, wall thickness changes, and alterations in contractility (7). For pressure overload-induced heart failure models, early echocardiographic measurements of LV mass and ejection fraction can predict later cardiac outcomes (8).

In the context of mouse heart transplantation, echocardiographic findings have been shown to correlate with graft function. Parameters such as ejection fraction and fractional shortening correlate closely with rejection and myocardial atrophy and high-resolution ultrasound provides information regarding the blood flow of transplanted mouse (9). In a small study, echocardiography has also been shown to reliably detect rejection (10). These advanced imaging techniques, combined with established genetic mouse models, have helped advance our understanding of graft rejection but require significant user expertise.

In this study, we focus on the use of M-mode echocardiography to assess graft viability and test the correlation between echocardiographic changes and the magnitude of the alloimmune responses. M-mode imaging provides a real-time, quantifiable, motion-based readout of contractile activity that straightforward to master. We hypothesized that echocardiography would outperform manual palpation in accurately defining rejection kinetics and would be a meaningful surrogate for the anti-donor T cell response.

## 2 Materials and methods

### 2.1 Study design and objective

This study compared manual palpation with trans-abdominal echocardiography (echo) in assessing graft function in a heterotopic heart transplant mouse model. The study consisted of three sequential phases: Phase 1 defined intra- and inter-operator differences in graft rejection determination when using manual palpation or echocardiography. Phase 2 assessed the diagnostic accuracy of each method using direct graft visualization as the gold standard. Phase 3 tested the correlation between echocardiographic findings and the magnitude of T cell alloimmune responses. Experiments in all phases were repeated once for confirmation.

### 2.2 Mice

WT C57BL/6J and WT BALB/c mice were bred at the Icahn School of Medicine at Mount Sinai (ISMMS) or purchased from the Jackson Laboratory (Bar Harbor, ME). Male and female mice were used in equal numbers as both recipients and donors. All animals were housed at the Icahn School of Medicine at Mount Sinai and cared for as per Institutional Animal Care and Use Committee (IACUC) and Association for Assessment and Accreditation of Laboratory Animal Care (AALAC) guidelines.

### 2.3 Antibodies and reagents

TONBO Bioscience: FC shield (cat. no. 70-0161-U500), CD4-PECy7 (cat. no. 60-0041-U100). BioLegend: MHC II–BV605 (cat. no. 107639), CD8–FITC (cat. no. 100706), H2-Kᵇ–PerCP-Cy5.5 (cat. no. 116516). Invitrogen: Viability–APC-Cy7 (cat. no. 65-0865-14), IFN*γ*–PerCP-Cy5.5 (cat. no. 45-7311-82), H2-Kᵈ–APC (cat. no. 17-5957-82), H2-Kᵇ–Pacific Blue (cat. no. 48-5958-82).

### 2.4 Mouse heterotopic heart transplantation

The microsurgery core at the ISMMS performed the heterotopic heart transplantation procedures as previously described (11). WT B6 mice between the ages of 6-12 weeks were used as recipients and treated with a single dose of 250ug CTLA4Ig (Abatacept) via intraperitoneal injection on post-transplant day 2. Both male and female mice were used as recipients. Buprenorphine (0.015 mg/mL) was administered subcutaneously (3.33uL/gm) for post-operative analgesia for five days post-transplant, in accordance with IACUC guidelines. All animals were housed under specific pathogen-free conditions.

### 2.5 Graft rejection assessment

Mice were assessed by both palpation and echocardiography on the same day but at independent times. All evaluators provided independent and blinded observations.

#### 2.5.1 Palpation

Two investigators independently assessed graft function by palpation of the abdomen starting on day 7 post-transplant and continuing 3 times per week until the end of each experiment. Rejection was defined as cessation of palpable beating on two consecutive evaluations. Palpator 1 (P1) was a post-doctoral fellow in a collaborating laboratory. Palpator 2 (P2) was a principal investigator in a separate collaborating laboratory. Both palpators had >3yrs of palpation experience.

#### 2.5.2 Echocardiography

Trans-abdominal echocardiography was performed on a Vevo 2100 High Resolution Imaging System (FUJIFILM VisualSonics, Toronto, Canada) using an MS550D (22-55 MHz) linear array transducer. The mice were anesthetized with 2% isoflurane and placed in a “spread-eagle” supine position on a heated platform during the evaluation. The parasternal long-axis view was used for image acquisition. M-mode imaging was employed to assess contractility. Imaging was collected on the same day as palpation assessment and evaluation of graft function was done by two blinded investigators. Based on pilot experiments using visual confirmation of cessation of heartbeat (data not shown), rejection was defined as either (1) absence of detectable contractile motion or (2) a beating frequency identical to the native heart (transmitted beat in a non-functional graft). At rejection of heterotopic hear transplants, the blood flow into the donor heart is interrupted leading to pulsations at the vascular anastomosis that get conducted through the graft and appears as rhythmic beating. This transmitted beat can be seen by echocardiography and felt during palpation. Echocardiographers had one assisted session with a Core facility ultrasonographer to familiarize themselves with appropriate use of the machine then three self-directed training sessions before performing all subsequent experiments independently.

Standardized imaging parameters:

- Frequency: 40 MHz
- Depth: 3 cm
- Gain: 28–35 dB
- Frame rate: 300–400 fps
- M-mode sweep speed: 100 mm/s
- Platform temperature: 37 °C

Visual and histological confirmation of rejection: After administration of high dose ketamine/xylazine anesthesia, laparotomy was performed and the transplanted heart was video recorded and evaluated for rejection (cessation of beating) by an independent investigator blinded to the findings of the palpators and ultrasonographers. Grafts were submitted for histologic analysis to confirm rejection in phase 2.

### 2.6 Cell isolation

Mouse spleens were physically dissociated, passed through a 40 μm strainer (Fisherbrand™ Sterile Cell Strainers, Thermo *Fisher Scientific, Waltham, MA, cat 22-363-547*), and lysed with RBC lysis buffer (*Thermo Fisher Scientific, Waltham, MA, cat A*1049201). To reduce experimental variability, all recipient splenocytes were frozen (90% FBS/10% DMSO) for synchronized testing in mixed lymphocyte reactions.

### 2.7 Mixed lymphocyte reaction

Frozen recipient splenocytes (responders) were defrosted simultaneously on the day of the experiment. Responder cells (2 × 10^6^) were co-cultured with allogeneic BALB/c antigen presenting cells (APCs, 2 × 10^6^) in RPMI media (Gibco, Thermo Fisher Scientific; cat. no. 11875-101) with 10% FBS (Gibco; cat. no. A5256801) as previously described (12). APCs were enriched from naïve BALB/c splenocytes by taking the *negative* fraction of the magnetic separation kit as per manufacturer's instructions (EasySep™ Mouse CD90.2 Positive Selection Kit II, Cat 18951, STEMCELL Technologies, Cambridge, MA). Co-cultures were incubated at 37 °C with 5% CO₂ for 24 h, followed by a 4-hour treatment with GolgiPlug (BD Biosciences; cat. no. 555029). Cells were then stained for viability and surface markers, fixed and permeabilized (eBioscience™ Fixation/Permeabilization Kit, Thermo Fisher Scientific; cat. no. 00-5523-00), stained for IFN*γ*, and analyzed by flow cytometry.

### 2.8 Statistical analysis

All statistical procedures were carried out with GraphPad Prism®, version 10.5.0 (GraphPad Software, San Diego, CA, USA). Continuous variables are expressed as mean ± standard deviation (SD) unless otherwise stated. Two-sided *p*-values < 0.05 were considered statistically significant. Normality was tested with the Shapiro–Wilk test and homogeneity of variances with Levene's test. For comparison between groups, normally distributed data were compared with an unpaired two-tailed Student's t-test; non-normal data were analyzed with the Mann–Whitney U test. Categorical comparisons, including the proportion of mice experiencing one or more discordant “reversal” episodes between serial graft assessments, were evaluated with a two-tailed Fisher's exact test; odds ratios (OR) and 95% confidence intervals (CI) were derived from the corresponding 2 × 2 contingency tables. For agreement between assessment methods, linear relationships between quantitative assessment techniques were quantified with the Pearson correlation coefficient (r) and its 95% confidence interval (CI). Diagnostic performance (sensitivity, specificity, positive/negative predictive values) was calculated for each method against the reference standard. Kaplan–Meier curves were constructed to estimate cumulative graft survival. Differences between curves were evaluated with the log-rank test.

## 3 Results

### 3.1 Echocardiography reduces operator-dependent experimental variability

**Figure 1:**
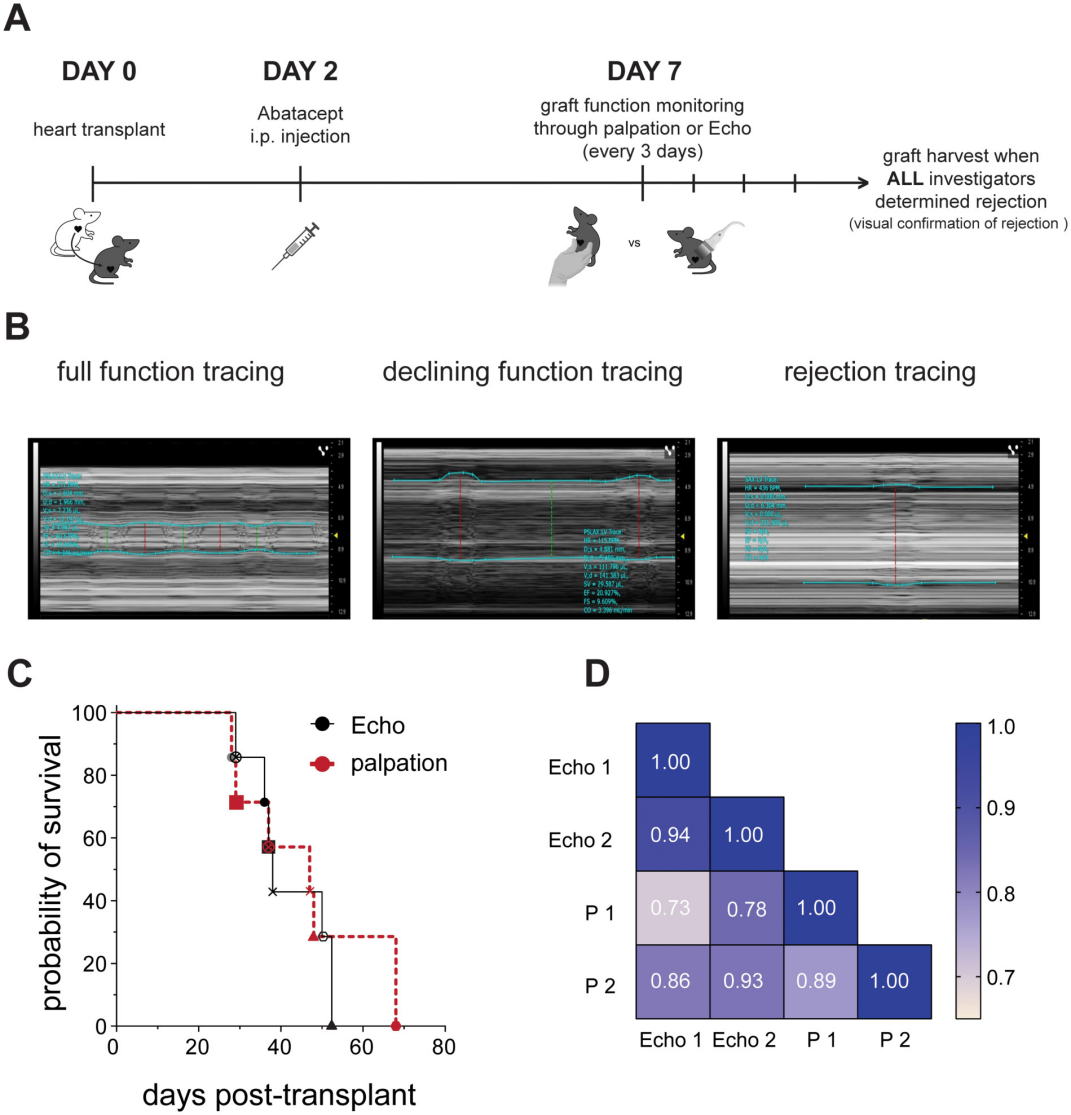
Reduced variability in delineating graft rejection using echocardiography. **(A)** Experimental schematic. **(B)** Representative tracing a full function (left), declining function (middle) and rejection (right). **(C)** Kaplan–Meier graft survival curves assessed by palpation (P, gray) and echocardiography (Echo, black). Each mouse was evaluated by both modalities. Matching symbols represent survival as determined by the two modalities (*n* = 6). **(D)** Pearson coefficient comparing reproducibility of graft rejection determination by the 4 blinded and independent observers (Echo 1, Echo 2, P 1, P 2).

We first compared echocardiography and manual palpation in determining rejection of cardiac allografts in mice. We transplanted a set (*n* = 6/group) of heterotopic BALB/c hearts into MHC-mismatched WT B6 mice treated with CTLA4Ig immunosuppression to delay rejection, then enlisted two blinded echocardiographers (Echo1 and Echo2) and two independent blinded palpators (P1 and P2) to assess graft rejection. To explicitly address the role of operator experience, palpation was performed by investigators with distinct training levels: an experienced investigator and a junior postdoctoral fellow early in training. This design intentionally reflects the structure of many academic laboratories, where graft monitoring is frequently performed by trainees and where variability introduced at this step can propagate through downstream analyses. The timing of rejection was reported to an independent team member who also sacrificed the mice for visual confirmation of graft rejection once all four evaluators recorded cessation of beating (Figure 1A, schematic).

At the cohort level, graft survival curves generated by palpation and echocardiography were broadly similar. However, rejection timing at the individual-animal level differed substantially between methods (Figures 1B–D). We quantified the concordance between each evaluator using Pearson's coefficient and found that Echo1 and Echo2 had nearly perfect concordance (Echo1 vs. Echo2: r = 0.94, 95% CI 0.51–0.99), while P1 and P2 had significant variation (P1 vs. P2: r = 0.89, 95% CI 0.29–0.98) (Figure 1D). When we compared echo with palpation, Palpator 2 (principal investigator, PI) had higher concordance with echocardiography than P1 (post-doctoral fellow), as anticipated.

We next evaluated the intra-observer variability between ultrasound and manual palpation. Due to the relative mobility of the transplanted heart and potential for overlying intestine interfering with assessment, determination of graft rejection requires serial palpations to confirm cessation of beating. In practice, a graft flagged as possibly rejected is often determined to be beating on subsequent evaluation. Manual palpation led to 42.6% of mice having at least one such discordant episode. By contrast, echocardiography was almost universally consistent in delineating rejection, with this form of discordance occurring only once (odds ratio = 0.01, *p* = 0.038 relative to manual palpation).

### 3.2 Enhanced sensitivity of echocardiography over palpation for determining graft function

**Figure 2:**
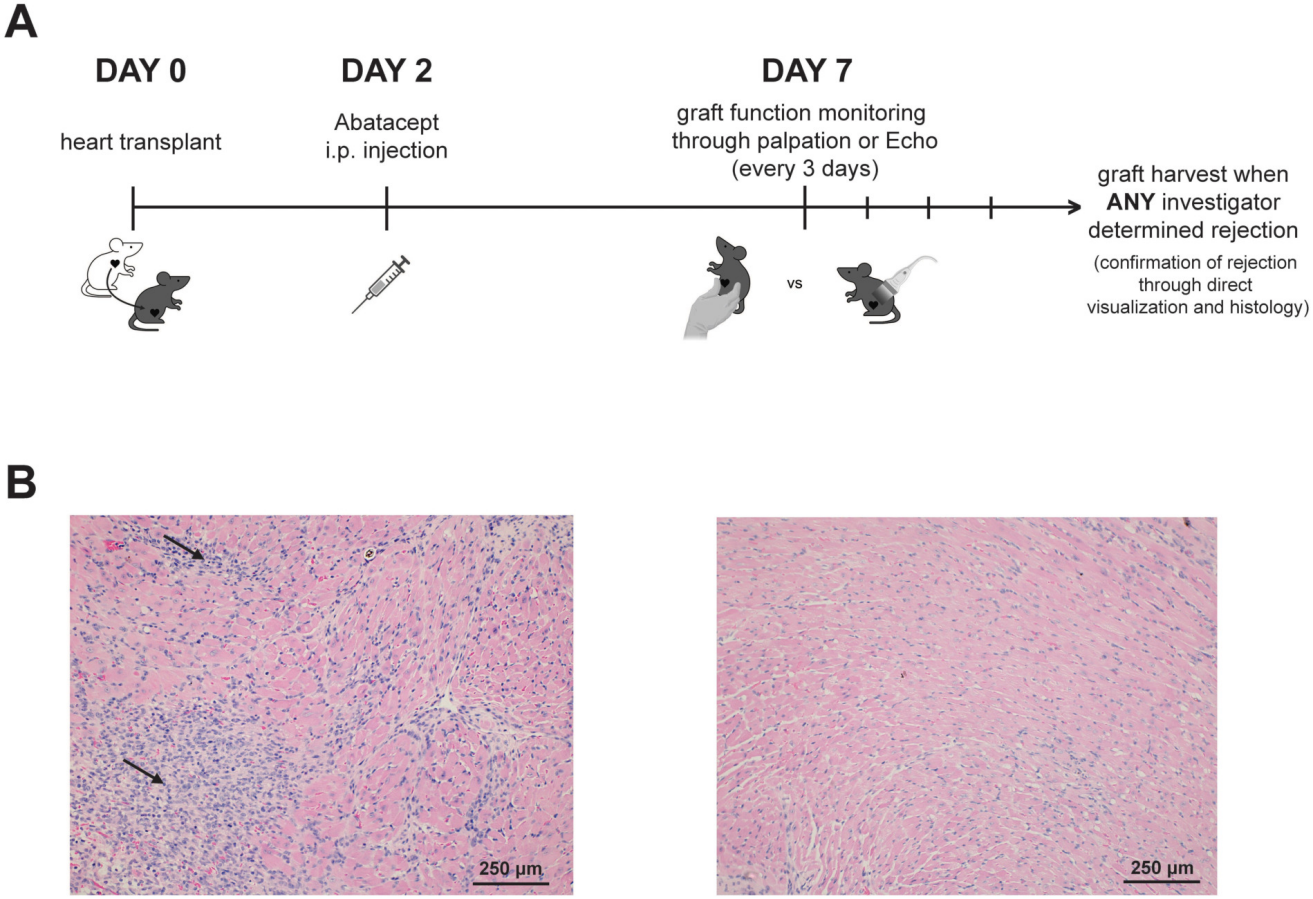
Improved accuracy of graft rejection diagnosis by echocardiography. **(A)** Experimental schematic. **(B)** Representative histologic images of true graft failure (left) and false graft failure (right) as determined by visual assessment of graft beating after laparotomy. Arrows indicate lymphocytic infiltrate.

To determine the accuracy of echo vs. palpation in determining graft rejection, we transplanted a second set of B6 mice with BALB/c hearts (*n* = 12 mice) and had the same two palpators and two echocardiographers evaluate the grafts for rejection. Here, we sacrificed the recipient when *any* evaluator determined graft rejection, and the heart was visually inspected after laparotomy for cessation of beating (gold standard) and submitted for additional histologic confirmation (Figures 2A,B). There were no differences in graft survival or M-mode trajectories when the recipients were stratified by sex (Supplementary Figure 1). Echocardiography achieved almost perfect diagnostic performance: sensitivity 92% (CI 64.4-98.5%), specificity 100% (CI 75.7%–100%), positive-predictive value (PPV) 100% (CI 74.1%–100%), negative-predictive value (NPV) 86% (CI 66.7–98.6%), and overall accuracy 96% (CI 79.8–99.3%) (average between the two investigators, Table 1). The composite sensitivity was derived from pooled data across both investigators: 11 true positives and 1 false negative across 12 total animals and the 95% confidence interval was calculated using the Wilson score method applied to the aggregate TP and FN counts. We show the individual investigator performance with separate confidence intervals in Table 1.

**Table 1:** Diagnostic performance of graft assessment methods.

	Echo 1	Echo 2	Palpator 1	Palpator 2
sensitivity	1 (CI 0.61–1)	0.83 (CI 0.44−0.97)	0.33 (CI 0.01–0.7)	0.5 (CI 0.19–0.81)
specificity	1 (CI 0.61–1)	1 (CI 0.61–1)	0.17 (CI 0.03–0.56)	0.83 (CI 0.44–0.97)
PPV	1 (CI 0.61–1)	1 (CI 0.57–1)	0.28 (CI 0.08–0.64)	0.75 (CI 0.3–0.95)
NPV	1 (CI 0.61–1)	0.86 (CI 0.49–0.97)	0.2 (CI 0.04–0.62)	0.625 (CI 0.3–0.86)
LR^+^	∞ (CI ∞-∞)	∞ (CI ∞-∞)	0.4 (CI 0.22–0.72)	3 (CI 1.44–6.24)
LR^−^	0 (CI 0–0.64)	0.17 (CI 0.03–0.92)	4 (CI 0.05–0.53)	0.6 (CI 0.19–1.86)
overall accuracy	1 (CI 0.76–1)	0.92 (CI 0.65–0.98)	0.27 (CI 0.1–0.57)	0.67 (CI 0.39–0.86)
true positive	6	5	2	3
true negative	6	6	1	5
false positive	0	0	5	1
false negative	0	1	4	3

Summary statistics for two independent investigators using echocardiography (Echo 1, 2) and two investigators performing graft palpation (Palpator 1, 2). Metrics are calculated against visual assessment of heartbeat cessation. An LR^+^ of ∞ indicates no false-positive results; an LR^−^ of 0 indicates no false-negative results. PPV, positive predictive value; NPV, negative predictive value; LR ^+^, positive likelihood ratio; LR^−^, negative likelihood ratio; CI, Confidence Interval; *N*, 12 mice.

Manual palpation again showed significant operator-dependent variability as previously. Palpator 1 detected 2 of the 6 true rejections (sensitivity 40%) and misclassified 5 of 6 beating grafts as rejected (specificity 17%), yielding an accuracy of 27% and a positive likelihood ratio (LR⁺) of 0.48, values akin to random chance. Palpator 2 performed better (sensitivity 50%, specificity 83%), but still misclassified one-third of examinations and produced a modest LR⁺ of 3 (Table 1). Taken together, these results confirm that high-resolution echocardiography provides more accurate detection of heterotopic heart-graft failure and effectively reduces operator-dependent variability. Indeed, significant palpator experience (post doc v PI) is required to overcome the improved performance from even limited training with echocardiography.

### 3.3 Echocardiography-guided assessment of alloimmunity reduces variability

**Figure 3:**
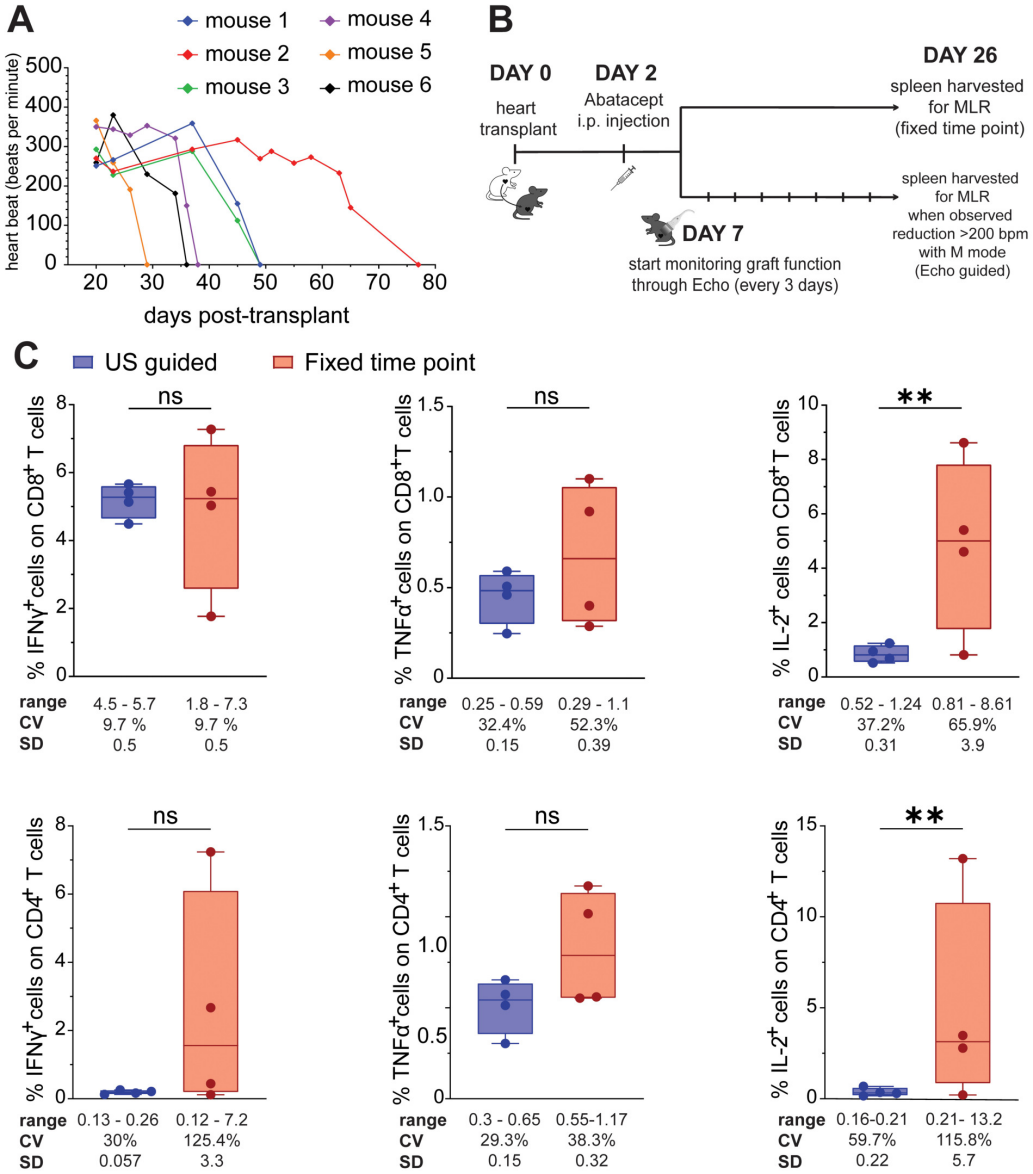
Echocardiography,guided assessment of alloimmune response reduces biologic variability. **(A)** Longitudinal post-transplant allograft heart rates assessed by echocardiography. **(B)** Experimental schematic. **(C)** Quantification and variance of IFN-*γ*, TNF-α, and IL-2 positive CD8 + and CD4+ T cells at a pre-specified post-transplant time point (Fixed time point) or at the time of a discrete change in allograft heart rate of 200 beats-per-minute as determined by echocardiography (Echo guided). *n* = 4/grp. CV = coefficient of variation, SD = standard deviation, by Welch's *t* test.

In transplant immunology studies, the magnitude of the anti-donor T cell response is quantified at a pre-determined post-transplant time-point, typically before rejections are predicted to occur. However, as the graft survival curves show (Figure 1C), the median time to rejection has significant variability, 42.5 days (IQR 29–55.5), suggesting that at any given time point the anti-donor immune reaction is similarly variable. A benefit in using echocardiography-guided evaluation of the allograft is direct visualization and measurement of allograft beating parameters. The M-mode, or motion mode, on ultrasound measures the movement of structures, such as the heart wall (Figure 1B). During phases 1 and 2 above, the echocardiographers noted that there was a distinct reduction in the M-mode (decrease of > 200 bpm) that preceded graft rejection. We verified this finding in a separate set of B6 recipient mice (*n* = 6) and observed that this change occurred in all mice at 10.3 +/- 4.8 days prior to rejection (Figure 3A).

We postulated that the observed change in M-mode on ultrasound correlated with the evolving magnitude of the allogeneic T cell immunity (T cells mediate rejection in this model). To test this, we transplanted two sets (*n* = 4/group) of WT B6 animals with BALB/c hearts as previously. For one set of mice, we quantified anti-donor T cell immunity by mixed lymphocyte reaction (MLR) at 26d post-transplant (usual practice, fixed time point), which was late post-transplant but before any grafts rejected. For the second set of mice, we harvested splenocytes for MLR when decrease in the M-mode as described above was recorded (Echo guided, Figure 3B, schematic).

The echo guided group had spleens harvested at 36.25 +/- 8.44 days post-transplant. The overall proportion of alloreactive IFN*γ*- and TNF*α*-producing CD4 + and CD8+ T cells did not differ between the two groups, while the percentage of IL2-producing T cell subsets was greater in the fixed time point group. Importantly, in all conditions, the variance in the alloreactive subsets was significantly lower (Welch's) in the echo guided than time point guided group (Figures 3C,D). Levene's test for homogeneity of variances also revealed a trend toward reduced variance in echo-guided assessment compared to fixed time-point, though did not statistical significance due to the small sample size (*n* = 4). These findings establish a link between alloimmune responses and quantifiable changes in graft function.

## 4 Discussion

The abdominal mouse heterotopic heart transplant model is an important tool in studying alloimmunity^2^. The ability to transplant defined inbred genetic strains as either recipient or donor in both allo- and syngeneic conditions has provided a unique platform to study both innate and adaptive alloimmune responses.

However, a known challenge in using the model is that manual palpation, the standard method for assessing graft viability, is operator dependent, prone to misclassification, and does not provide information regarding with the intensity of anti-donor immunity, which poses limitations on the translational potential of the model. Comparing M-mode transabdominal echocardiography to manual palpation, we established that echocardiography more accurately delineated cessation of graft beating and reduced intra-observer variability (Figures 1, 2). Importantly, we found that ultrasonography removed the ambiguity inherent in palpation assessments of graft rejection.

We also newly showed that a reduction in the allograft contractility (detected by M-mode ultrasonography) correlated with the magnitude of the anti-donor T cell response (Figure 3). Ultrasound guided assessment of alloreactivity significantly reduced the experimental variability of immune assays compared to testing at pre-specified time points. We did find a statistically significant increase in IL2-producing cells in the fixed-time point compared to echo-guided group, supporting our hypothesis that fixed-time point analysis captures a broad and heterogenous stage of alloimmune activation. We saw no sex differences (data not shown) in the immunologic or graft rejection kinetics. The use of blinded investigators and lab members from various transplant research labs strengthens the rigor and applicability of these findings, respectively.

These data have important practical significance for experimental design. The increased accuracy of echocardiography over manual palpation in delineating graft rejection can improve the rigor and reproducibility of survival experiments. While the survival curves in Figure 1 overlap, closer inspection shows that “rejection” for each individual mouse varies up to 16d between palpation and echocardiography, consistent with reduced sensitivity of the former.

Additionally, immune assays such as the MLR are important readouts in transplant studies but require sacrificing the recipient animal. Using M-mode changes as a surrogate for T cell alloreactivity could significantly decrease the number of animals required in pilot experiments (i.e., T cell response can be assessed in the same mice used in survival kinetic experiments) and simultaneously provide guidance on when to perform T cell assays in future experiments. We selected the 200bpm drop in graft heart rate based on its association with graft survival (Figure 2), but future studies may identify alternative parameters (e.g., percentage changes from baseline or relative to native heart rate) that similarly reduce T cell assay variability and/or correlate with more rigorous histologic analysis (e.g., International Society for Heart and Lung Transplantation, ISHLT, grading) of damage and rejection.

While testing the effect of pharmacologic interventions is beyond the scope of this study, future work using changes detected by echocardiography to guide timing of drug administration may be the most consequential impact of M-mode based allograft ultrasonography. In clinical practice, interventions are guided by changes in clinical parameters (e.g., reduction in organ function, biopsy diagnosis of rejection) and not at pre-specified post-transplant time point. Graft echocardiography aligns the pre-clinical model more closely with care of human patients and may increase the translational utility of this model.

There are some limitations to imaging-based analysis. Echocardiography requires access to specific equipment, sedation of the animals, and is more time-consuming than manual palpation. At our institute, we have a Biomedical imaging Core facility that maintains a clinical-grade ultrasound machine and provides technical training for reproducible imaging. Ultrasound training itself was straightforward and took only 4 sessions (1 technician-guided, 3 independent) to achieve proficiency. While we do not have a direct record to compare for palpation, it typically takes new trainees several months or more of consistent palpation experience to gain proficiency in our labs. We anticipate that with increasing availability of user-friendly imaging platforms [e.g., point-of-care ultrasound (13)], echocardiography-guided monitoring may become easier to integrate into routine post-transplant monitoring protocols. Need for sedation also limited our evaluations to three times per week, rather than daily. We did not specifically study the effects of repeated isoflurane anesthesia affected graft outcome, but do not anticipate there was a significant impact based on published graft outcome kinetics (6, 12).

Taken together, these data indicate that manual palpation of graft survival provides a reasonable assessment of graft survival, supporting its current use. However, inexperienced palpators introduce substantial variability that reduces statistical power and increases animal usage. Incorporation of echocardiography offers a practical strategy to improve rigor, enhance reproducibility, and reduce animal numbers, and supports further studies to validate echocardiography as a non-invasive surrogate for anti-donor T cell immunity.

## Data Availability

The original contributions presented in the study are included in the article/Supplementary Material, further inquiries can be directed to the corresponding author.
