# Supplementary material for: Echocardiography-guided assessment of mouse cardiac transplant rejection improves model reproducibility
Source: Front Transplant. 2026 Jun 17;5:1840060. doi: 10.3389/frtra.2026.1840060 (PMC13318921; doi:10.3389/frtra.2026.1840060)
Supplement: Supplementary file 1 [file Datasheet1.pdf]

A

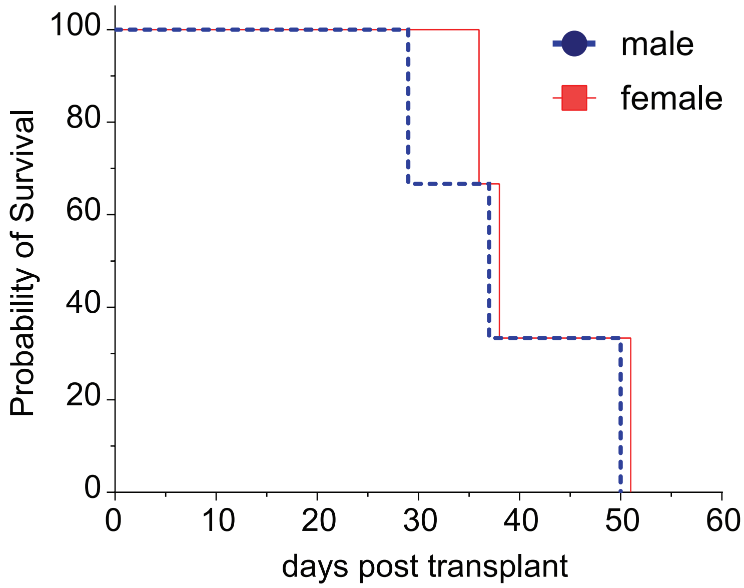

Supplemental figure 1. Graft survival stratified by sex. Kaplan-Meier graft survival curves assessed by echocardiography stratified by sex show no differences in graft survival between sexes (3 males and 3 females mice, phase 1).
